# High-Throughput Untargeted Serum Metabolomics Analysis of Hyperuricemia Patients by UPLC-Q-TOF/MS

**DOI:** 10.1155/2021/5524772

**Published:** 2021-06-12

**Authors:** Nankun Qin, Yue Jiang, Wenjun Shi, Liting Wang, Lingbo Kong, Chengxiang Wang, Yuying Guo, Jiayu Zhang, Qun Ma

**Affiliations:** ^1^School of Chinese Pharmacy, Beijing University of Chinese Medicine, Beijing 102488, China; ^2^Beijing University of Chinese Medicine Affiliated Dongzhimen Hospital, Beijing 100010, China; ^3^School of Pharmacy, Binzhou Medical University, Yantai 264003, China

## Abstract

Hyperuricemia (HUA) as a metabolic disease is closely associated with metabolic disorders. The etiology and pathogenesis of HUA are not fully understood, so there is no radical cure so far. Metabolomics, a specialized study of endogenous small molecule substances, has become a powerful tool for metabolic pathway analysis of selected differential metabolites, which is helpful for initially revealing possible development mechanisms of various human diseases. Twenty HUA patients and 20 healthy individuals participated in the experiment, and ultrahigh performance liquid chromatography coupled with quadrupole time-of-flight tandem mass spectrometry (UPLC-Q-TOF/MS) was employed to investigate serum samples to find differential metabolites. The statistical techniques used were principal component analysis and orthogonal partial least-squares discriminant analysis. The differences in metabolomics results of samples after pretreatment with different solvents were compared, 38, 20, 26, 28, 33, 50, and 40 potential differential metabolites were found, respectively, in HUA patient samples, and each group involved different metabolic pathways. Repetitive metabolites were removed, 138 differential metabolites in HUA serum were integrated for analysis, and the human body was affected by 7 metabolic pathways of glycerophospholipid metabolism, sphingolipid metabolism, arachidonic acid metabolism, linoleic acid metabolism, phenylalanine metabolism, phenylalanine, tyrosine and tryptophan biosynthesis, and *α*-linolenic acid metabolism. In this work, the metabolomics approach based on UPLC-Q-TOF/MS was employed to investigate serum metabolic changes in HUA patients, 138 potential differential metabolites related to HUA were identified, which provided associations of lipids, amino acids, fatty acids, organic acids, and nucleosides profiles of HUA individuals. Metabolic pathways involved in glycerophospholipid metabolism, sphingolipid metabolism, arachidonic acid metabolism, linoleic acid metabolism, phenylalanine metabolism, phenylalanine, tyrosine and tryptophan biosynthesis, and *a*-linolenic acid metabolism shed light on the understanding of the etiology and pathogenesis process of HUA.

## 1. Introduction

Hyperuricemia (HUA) as a metabolic disease is closely associated with metabolic disorders and is an important risk factor for gout, cardiovascular disease, metabolic syndrome, and so on [1–6]. In recent years, with the improvement of living standards, more high-purine, high-protein, and high-calorie foods have entered people's diet structure [7]; thus, the prevalence of HUA is on the rise globally with the trend of younger age [8–14]. The etiology and pathogenesis of HUA are not fully understood, so there is no radical cure so far. Therefore, it is of great significance to explore metabolic disorders in vivo and the pathogenesis of HUA from the perspective of metabolomics.

Metabolomics analysis explores the dynamic changes of endogenous small molecule substances (saccharides, organic acids, lipids, amino acids, etc.) at a certain stage of the disease [15]. Metabolomics has been widely applied in the field of human healthy metabolic pathway analysis of selected differential metabolites and is helpful for initially revealing the possible development mechanisms of various human diseases [16, 17]. The study of metabolic disorders is meant for assisting disease, clinical diagnosis, screening of therapeutic drugs and targets [18–20]. Over recent years, extensive studies have been extensively conducted on HUA metabolomics, but these studies were mainly focused on animal experiments and there is little research describing HUA metabolic disorders in humans [21–25].

The integrity of the extraction of endogenous small molecular substances from biological samples is tremendously important for the analysis results. The loss of biological information caused by improper extract methods has considerably affected the elucidation of disease mechanisms [26–28]. Organic solvent protein precipitation (PPT) is simple, economical, and easy to perform that has usually been regarded as the most commonly used extract method for serum samples prior to metabolomics analysis [29, 30]. Precipitation solvents often adopted include methanol (MeOH) or acetonitrile (MeCN) and a mixture of two [31–33]. We have simply analyzed the relationship between several precipitation solvents of PPT and the analysis results and subsequently recommend appropriate protocols of serum preparation for HUA metabolomics analysis.

In this study, high-sensitivity and high-resolution ultrahigh performance liquid chromatography coupled with quadrupole time-of-flight tandem mass spectrometry (UPLC-Q-TOF/MS) combined with multivariate statistical analysis was applied; the metabolome of HUA patients was investigated to identify serum differential metabolites and analyze metabolic pathways affected by HUA, exploring its mechanisms.

## 2. Materials and Methods

### 2.1. Reagents and Instruments

Liquid chromatography-mass spectrometry (LC-MS) grade MeOH and MeCN were purchased from Merck (Darmstadt, Germany). Ultrapure water was purified by a Milli-Q water system (Millipore, Milford, MA, USA). LC-MS grade formic acid was obtained from Fisher Scientific Corporation (Loughborough, UK).

Instruments used in this study include vortex mixer (Haimen Kylin-Bell Lab Instruments Co., Ltd., Jiangsu, China), cryogenic supercentrifuge (Shanghai Luxiang Instrument Co., Ltd., Shanghai, China), nitrogen evaporator (Beijing Chengmeng Weiye Technology Co., Ltd., Beijing, China), and UPLC-Q-TOF/MS (Waters Corp., Milford, MA, USA).

### 2.2. Participants

Participants were collected from the Rheumatology Clinic and Physical Examination Center of Beijing University of Chinese Medicine Affiliated Dongzhimen Hospital (Beijing, China). HUA patients (*n* = 20) and healthy volunteers (*n* = 20) were enrolled in this study. The related clinical information, including gender, age, and biochemical indexes of serum, was collected. Inclusion criteria were as follows: (1) serum uric acid level was ≥420 *μ*mol/L in males and ≥360 *μ*mol/L in females and (2) aged between 20 and 65 years. Exclusion criteria were as follows: (1) pregnant or lactating women; (2) suffering from cardiovascular disease, kidney disease, or other diseases that will affect the clinical observations and biological indicators or having metabolic diseases, tumors, and mental disease; (3) patients with HUA caused by the following drugs: thiazide diuretics, furosemide, pyrazinamide, aspirin, and other drugs. These participants did not take medicines or supplements before they collected serum samples. Verbal informed consent from all subjects was obtained, and the project was approved by the Ethics Committee of Beijing University of Chinese Medicine Affiliated Dongzhimen Hospital and was conducted according to the Declaration of Helsinki Principles. All serum samples were stored at −80°C before analysis.

### 2.3. Sample Preparation

The same batch of serum samples were pretreated by seven organic solvent protocols (MeOH, MeOH-MeCN (90 : 10, v/v), MeOH-MeCN (70 : 30, v/v), MeOH-MeCN (50 : 50, v/v), MeOH-MeCN (30 : 70, v/v), MeOH-MeCN (10 : 90, v/v), and MeCN) labelled as groups A, B, C, D, E, F, and G, respectively. Frozen serum samples, including 20 HUA samples (HUA group) and 20 healthy samples (control group), were thawed at 4°C; then, each sample was divided into seven aliquots of 100 ul. Then, 300 *µ*L of the corresponding organic solvent was added to each 100 *µ*l serum aliquot, vortexed for 5 min, and incubated for 10 min on ice; it was then centrifuged at 12 000 r/min for 10 min at 4°C. All supernatants were evaporated to dryness. Afterwards, the residues were reconstituted in 100 *µ*L of 80% MeOH aqueous, vortexed for 5 min, and incubated for 10 min on ice; then, they were centrifuged at 12 000 r/min for 10 min at 4°C. The supernatant was analyzed using UPLC-Q-TOF/MS.

### 2.4. UPLC-Q-TOF/MS Conditions

The chromatographic separation was achieved on an Acquity UPLC^TM^ System coupled to a Xevo G2 Q-TOF/MS with a Waters UPLC BEH C18 column (2.1 × 100 mm I.D., 1.7 *µ*m; Waters Corp., Milford, MA, USA) at a column temperature of 45 °C. The mobile phase was composed of 0.2% formic acid aqueous solution (A) and MeOH (B) with the gradient set as follows: 0–1.0 min, 95–95% B; 1.0–2.0 min, 95–2% B; 2.0–13.0 min, 2–2% B; 13–13.5 min, 2–95% B; 13.5–15 min, 95–95% B. The flow rate was 0.40 mL/min, and the injection volume was 2 *μ*L. The autosampler temperature was conditioned at 4°C.

Electrospray ionization (ESI) in positive ion (ESI+) mode and negative ion (ESI−) mode was applied for high-resolution MS detection. The mass range was set at m/*z* 50–1200 Da. The optimized operating parameters were set as follows: ion spray voltage of 3.0 kV, cone voltage of 25 V, cone gas flow of 50 L/h, source temperature of 120 °C, dry gas (N_2_) flow of 10 mL/min, atomization temperature of 450 °C, and 400 °C for ESI+ and ESI−. MS data were recorded in MSE mode. The accurate mass and composition of the relative target ions were calculated with MassLynx V 4.0 software (Waters Corp., Milford, MA/USA).

### 2.5. Data Processing and Multivariate Data Analysis

Raw data from the seven protocols were processed by Progenesis QI software (Nonlinear Dynamics, Newcastle upon Tyne, UK) for peak detection, peak alignment, normalization, and other operations. Finally, two-dimensional data matrices, including the m/*z* value, retention times, and normalized peak area, were generated. These two-dimensional data matrices were, respectively, imported into SIMCA-P 14.1 software (Umetrics AB, Umea, Sweden) for pattern recognition. Principal component analysis (PCA) revealed the distribution of metabolites in human serum samples. Orthogonal partial least-squares discriminant analysis (OPLS-DA) models were constructed to distinguish sample differences and mine differential metabolites in massive data. Permutation tests were used to verify the validity of the OPLS-DA model. The contribution rate of a variable is often described by the variable importance of the projection (VIP) value. The greater the contribution rate is, the larger the VIP value is. The VIP values were generated by the OPLS-DA model. Metabolites with VIP greater than 1, *p* values of *t*-test (*p*) less than 0.05, and a fold change (FC) of greater than 2.0 or FC less than 0.5 were selected as differential metabolites.

### 2.6. Metabolites Identification and Metabolic Pathway

The chemical information of differential metabolites was searched through the human metabolome database (HMDB; http://www.hmdb.ca/) and METLIN (http://metlin.Scripps.edu). Input the precise molecular mass, ionization method, and addition ion information of differential metabolites into HMDB and METLIN, in accordance with the rule that the deviation of the m/*z* value does not exceed 0.02. The identification results are proved by combining the exact number of charges and the ionization method that meets the experimental conditions. Compare the primary and secondary mass spectra information of the differential metabolites with the theoretical fragments of the HMDB search results, then infer the structure of the compound and the attribution of the fragments to obtain the HUA differential metabolites.

Moreover, for exploring how the major metabolic pathways related to the differential metabolites were affected, metabolic pathway analysis was performed by MetaboAnalyst 5.0 [34] platform (http://www.metaboanalyst.ca). All metabolic pathways found displayed their impact values and *p* values in the form of bubbles. The metabolic pathways with a pathway impact of >0.2 and *p* < 0.05 were considered the most significant.

## 3. Results and Discussion

### 3.1. Basic Characteristics and Biochemistry Results

Basic characteristics and serum biochemistry results of participants in the control group and HUA group are presented in Table 1. The control group included 20 participants, with a mean age of 40.3 ± 11.6 years; 55% were male. The mean age of 20 participants in the HUA group was 41.1 ± 12.6 years; 55% were male. There was no significant difference (*p* > 0.05) between age and gender. Compared with the control group, fasting serum glucose (FSG), uric acid (UA), triglyceride (TG), alanine aminotransferase (ALT), aspartic aminotransferase (AST), high-density lipoprotein cholesterol (HDL), low-density lipoprotein cholesterol (LDL), and creatinine (CR) of the patients in the HUA group all increased significantly (*p* < 0.05).

### 3.2. Chromatographic Analysis and Comparison between the Seven Groups

**Figure 1:**
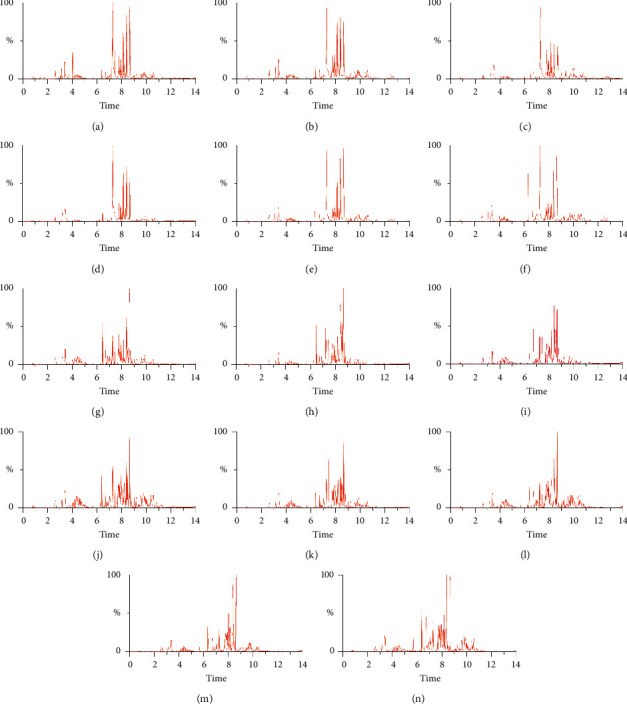
Typical UPLC-Q-TOF/MS base peak ion (BPI) chromatograms of serum metabolite profiles from each group in ESI + mode. BPI chromatograms of the control group (a) and HUA group (b) from group A. BPI chromatograms of the control group (c) and HUA group (d) from group B. BPI chromatograms of the control group (e) and HUA group (f) from group C. BPI chromatograms of the control group (g) and HUA group (h) from group D. BPI chromatograms of the control group (i) and HUA group (j) from group E. BPI chromatograms of the control group (k) and HUA group (l) from group F. BPI chromatograms of the control group (m) and HUA group (n) from group G.

**Figure 2:**
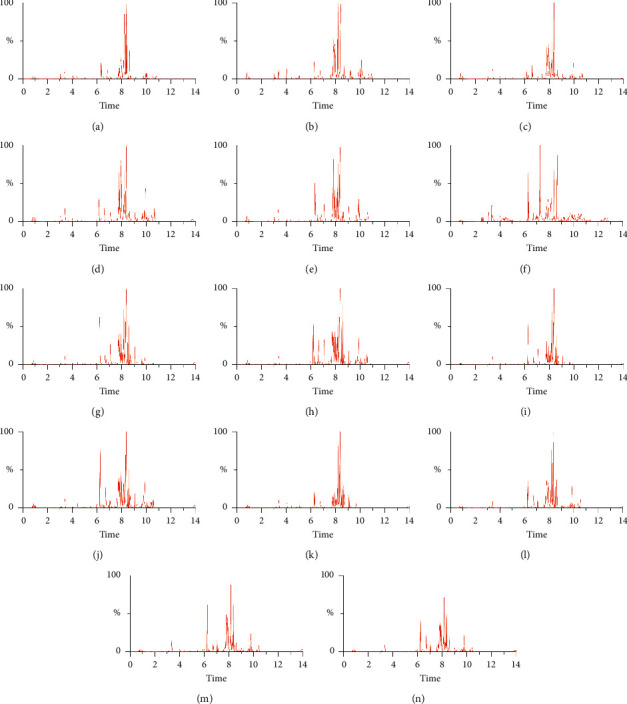
Typical UPLC-Q-TOF/MS BPI chromatograms of serum metabolite profiles from each group in in ESI− mode. BPI chromatograms of the control group (a) and HUA group (b) from group A. BPI chromatograms of the control group (c) and HUA group (d) from group B. BPI chromatograms of the control group (e) and HUA group (f) from group C. BPI chromatograms of the control group (g) and HUA group (h) from group D. BPI chromatograms of the control group (i) and HUA group (j) from group E. BPI chromatograms of the control group (k) and HUA group (l) from group F. BPI chromatograms of the control group (m) and HUA group (n) from group G.

Base peak ion chromatograms in ESI + mode and ESI− mode from seven HUA groups and corresponding control groups were compared as shown in Figures 1 and 2. Differences in peak numbers and heights were observed between the HUA group and the control group, which indicated that the composition and content of metabolites in humans in different physiological states were different. In addition, the HUA group and the control group showed different metabolic profiles after being treated with different pretreatment solvents.

### 3.3. Multivariate Statistical Analysis

**Figure 3:**
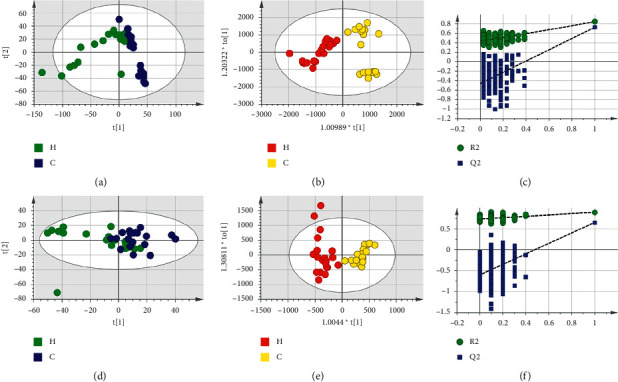
Group A's PCA score plot, OPLS-DA score plot, and permutation test plot of a serum sample from the control group and HUA group in ESI + mode and ESI− mode. (a, d) The PCA score plots in ESI + mode and ESI− mode, respectively. (b, e) The OPLS-DA score plots in ESI + mode and ESI− mode, respectively. (c, f) Permutation test plots in ESI + mode and ESI− mode, respectively. C: control group; H: HUA group.

**Figure 4:**
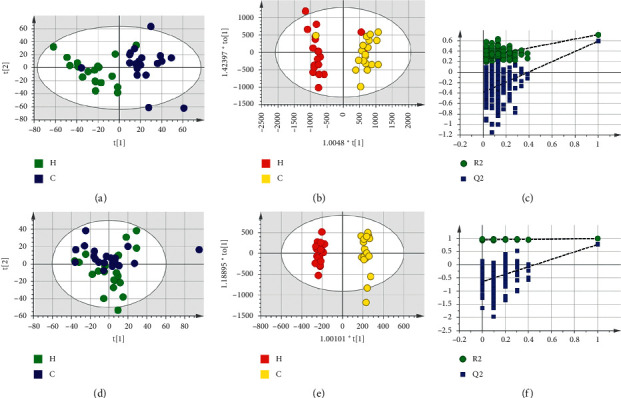
Group B's PCA score plot, OPLS-DA score plot, and permutation test plot of a serum sample from the control group and HUA group in ESI + mode and ESI− mode. (a, d) The PCA score plots in ESI + mode and ESI− mode, respectively. (b, e) The OPLS-DA score plots in ESI + mode and ESI− mode, respectively. (c, f) Permutation test plots in ESI + mode and ESI− mode, respectively. C: control group; H: HUA group.

**Figure 5:**
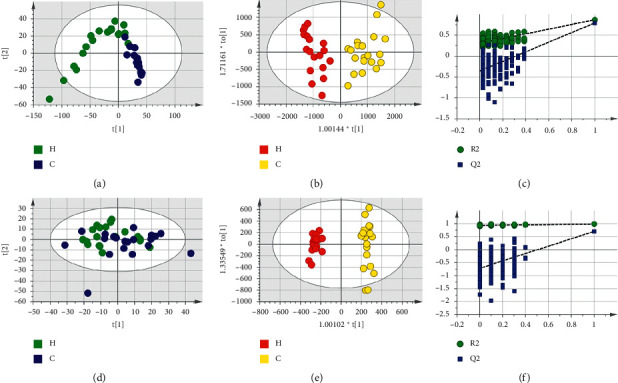
Group C's PCA score plot, OPLS-DA score plot, and permutation test plot of a serum sample from the control group and HUA group in ESI + mode and ESI− mode. (a, d) The PCA score plots in ESI + mode and ESI− mode, respectively. (b, e) The OPLS-DA score plots in ESI + mode and ESI− mode, respectively. (c, f) Permutation test plots in ESI + mode and ESI− mode, respectively. C: control group; H: HUA group.

**Figure 6:**
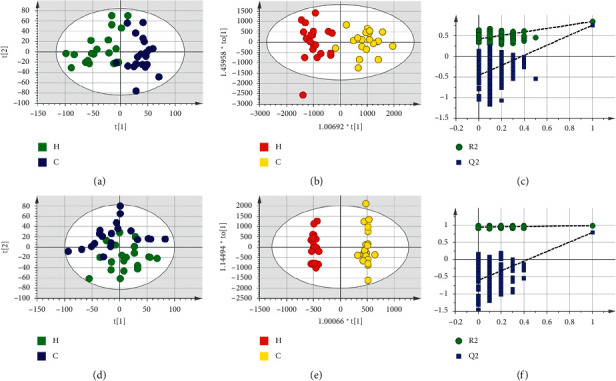
Group D's PCA score plot, OPLS-DA score plot, and permutation test plot of a serum sample from the control group and HUA group in ESI + mode and ESI− mode. (a, d) The PCA score plots in ESI + mode and ESI− mode, respectively. (b, e) The OPLS-DA score plots in ESI + mode and ESI− mode, respectively. (c, f) Permutation test plots in ESI + mode and ESI− mode, respectively. C: control group; H: HUA group.

**Figure 7:**
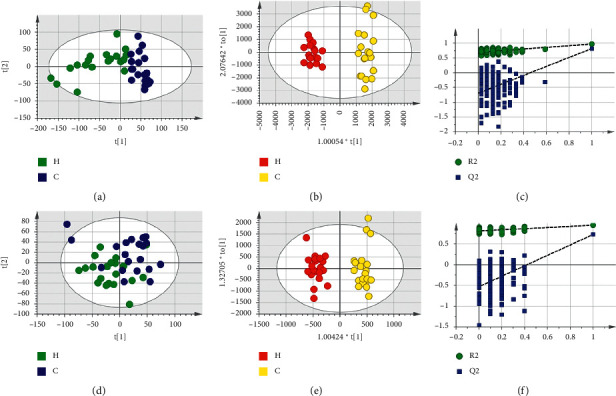
Group E's PCA score plot, OPLS-DA score plot, and permutation test plot of a serum sample from the control group and HUA group in ESI + mode and ESI− mode. (a, d) The PCA score plots in ESI + mode and ESI− mode, respectively. (b, e) The OPLS-DA score plots in ESI + mode and ESI− mode, respectively. (c, f) Permutation test plots in ESI + mode and ESI− mode, respectively. C: control group; H: HUA group.

**Figure 8:**
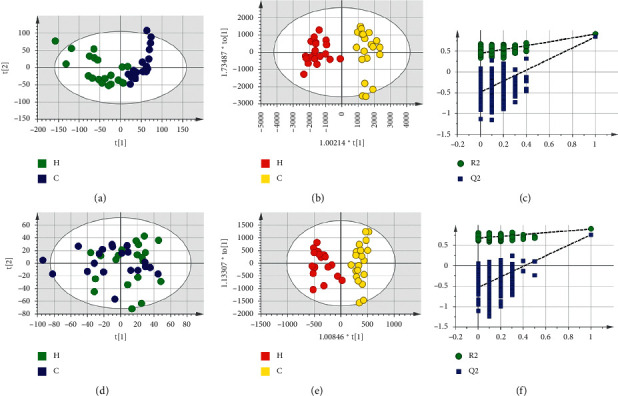
Group F's PCA score plot, OPLS-DA score plot, and permutation test plot of a serum sample from the control group and HUA group in ESI + mode and ESI− mode. (a, d) The PCA score plots in ESI + mode and ESI− mode, respectively. (b, e) The OPLS-DA score plots in ESI + mode and ESI− mode, respectively. (c, f) Permutation test plots in ESI + mode and ESI− mode, respectively. C: control group; H: HUA group.

**Figure 9:**
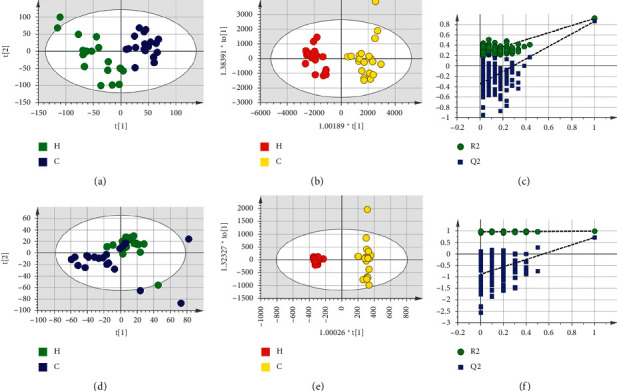
Group G's PCA score plot, OPLS-DA score plot, and permutation test plot of a serum sample from the control group and HUA group in ESI + mode and ESI− mode. (a, d) The PCA score plots in ESI + mode and ESI− mode, respectively. (b, e) The OPLS-DA score plots in ESI + mode and ESI− mode, respectively. (c, f) Permutation test plots in ESI + mode and ESI− mode, respectively. C: control group; H: HUA group.

Based on LC-MS results of seven groups' serum samples, PCA was used to study the distribution of metabolites. Figures 3(a)–9(a) show the seven groups' PCA score plots of the control group and HUA group in ESI + mode. Figures 3(d)–9(d) show seven groups' PCA score plots of the control group and HUA group in ESI− mode. According to the PCA score plots, the metabolic patterns of humans behaved differently in different statuses. It revealed that HUA would cause disturbance in the metabolic pathway in humans. There were many influencing factors for the clinical samples, such as gender, age, region, diet, and living environment, which caused considerable noise signals unrelated to grouping the information. Thus, PCA appeared partially overlapped and therefore cannot be further interpreted.

The OPLS-DA model showed the differences between the HUA group and control group more clearly compared with the results of PCA. Seven groups' OPLS-DA score plots of the control group and HUA group in ESI + mode were shown in Figures 3(b)–9(b). Seven groups' OPLS-DA score plots of the control group and HUA group in ESI− mode were shown in Figures 3(e)–9(e). Except for OPLS-DA score plot of group B in ESI + mode, the HUA group in each OPLS-DA score plots showed an obvious separation trend from the corresponding control group, which means there was a significant difference in metabolic profiles between the two groups. The values of *R*^2^*Y* and *Q*^2^ of the OPLS-DA model listed in Table 2 were higher than 0.593, showing that the established model had a high stability and prediction rate. The permutation test (*n* = 200) was further used to validate the model, and Figures 3(c)–9(c) and Figures 3(f)–9(f) are the results of the permutation tests of seven groups. All *R*^2^ and *Q*^2^ values were smaller than the values in the actual model, indicating that there was no overfitting in the OPLS-DA model.

Furthermore, the metabolic patterns of human behaved differently in different groups according to the PCA and OPLS-DA score plots. It revealed that the pretreatment solvent would affect the results revealing the disorder of the metabolic pathway in human.

### 3.4. Metabolite Identification and Metabolic Pathway

There were, respectively, 296 (Group A), 260 (Group B), 150 (Group C), 203 (Group D), 393 (Group E), 461 (Group F), and 382 (Group G) differential metabolites between the control group and HUA group satisfying VIP > 1.0, *p* < 0.05, and FC > 2.0 or FC < 0.5. According to the online database, 38 (Group A), 20 (Group B), 26 (Group C), 28 (Group D), 33 (Group E), 50 (Group F), and 40 (Group G) characteristic metabolites in patient serum metabolic profiles of seven groups were finally identified, and the results are listed in Tables S1-S7.

**Figure 10:**
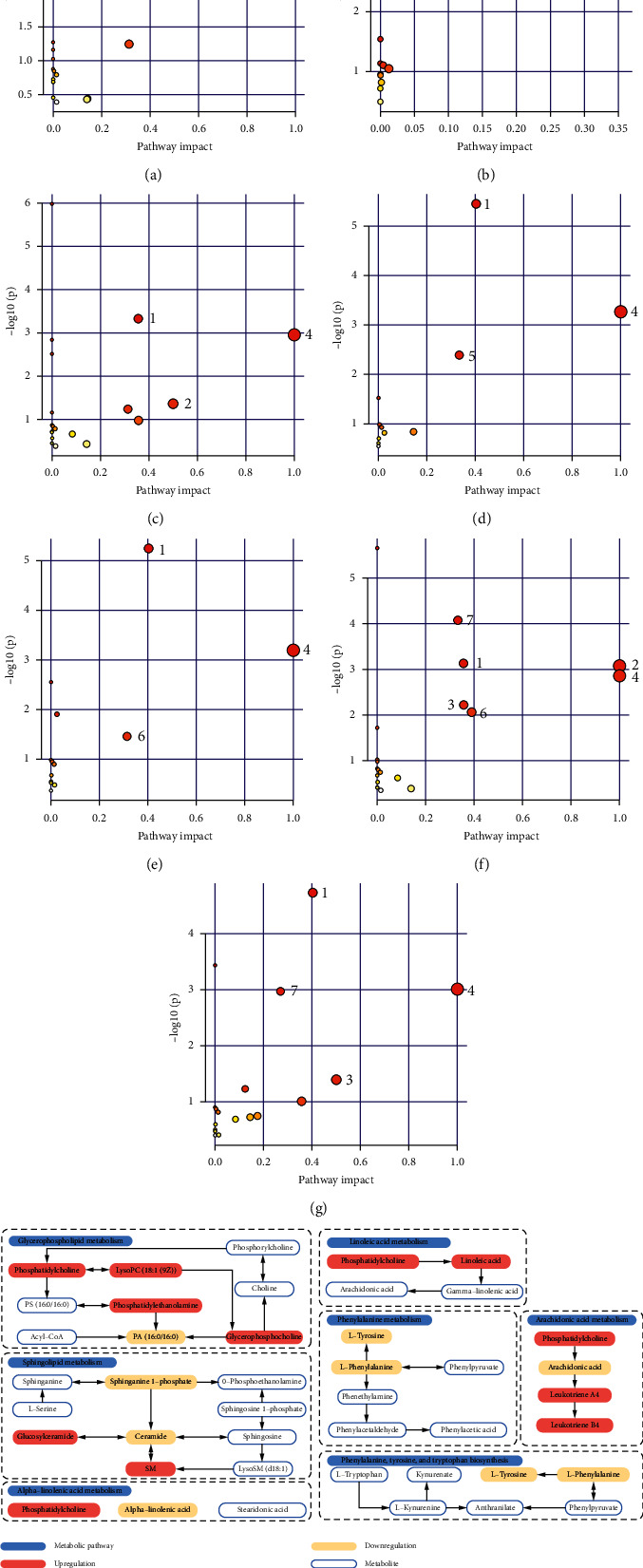
Metabolic pathway analysis of seven groups. Metabolic pathway analysis of (a) Group A identified metabolites, (b) Group B identified metabolites, (c) Group C identified metabolites, (d) Group D identified metabolites, (e) Group E identified metabolites, (f) Group F identified metabolites, and (g) Group G identified metabolites. (h) A schematic diagram of the relevant metabolic pathways. 1: glycerophospholipid metabolism; 2: phenylalanine, tyrosine, and tryptophan biosynthesis; 3: phenylalanine metabolism; 4: linoleic acid metabolism; 5: *a*-linolenic acid metabolism; 6: arachidonic acid metabolism; 7: sphingolipid metabolism.

The significant 35 metabolic pathways of serum differential metabolites from the seven groups are shown in Figures 10(a)–10(g). The result implied that multiple metabolic pathways had a certain extent of disturbance effect on HUA. The relevant metabolic pathways for the seven groups are numerically labelled in the figure. The results suggested that the seven groups of differential metabolites mainly involved the seven metabolic pathways of glycerophospholipid metabolism, sphingolipid metabolism, arachidonic acid metabolism, linoleic acid metabolism, phenylalanine metabolism, phenylalanine, tyrosine and tryptophan biosynthesis, and *a*-linolenic acid metabolism. A schematic diagram of the relevant metabolic pathways was summarized in Figure 10(h).

## 4. Discussion

HUA is a complex metabolic syndrome, which is the result of a combination of multiple factors [35–39], including gender, age, heredity, metabolism, environment, diet, race, drug, and disease, and may also be related to other potential factors. A study of HUA patients and healthy people by metabolomics techniques is conducted to find the change rules from the complex endogenous metabolites of HUA and to explore the etiology and pathogenesis of HUA.

The pretreatment solvents, MeOH, MeOH-MeCN (90 : 10, v/v), MeOH-MeCN (70 : 30, v/v), MeOH-MeCN (50 : 50, v/v), MeOH-MeCN (30 : 70, v/v), and MeOH-MeCN (10 : 90, v/v), and pathogenesis MeCN were compared; except for the solvents used, the subsequent operations were the same. LC-MS/MS is one of the most commonly used methods for metabolite analysis of biological samples in metabolomics analysis. In this study, a UPLC-Q-TOF/MS-based serum metabolomics approach coupled with multivariate statistical analysis provided a convincing method to clearly differentiate patients with HUA from matched control subjects and identify the potential differential metabolites. Results indicate that OPLS-DA revealed an evident separation between the HUA and control samples. The number and types of differential metabolites in the seven groups are not the same, and the metabolic pathways involved are also different, which will have an impact on the reference direction for exploring the HUA mechanism. If a serum precipitation solvent suitable for HUA metabolomics analysis must be recommended, then MeOH–MeCN (10 : 90, v/v) can be used for subsequent analysis.

**Figure 11:**
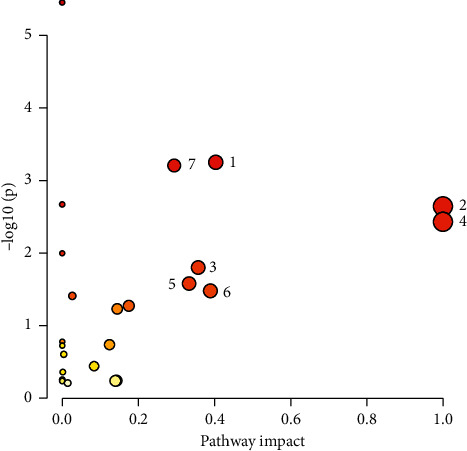
Metabolic pathway analysis of 138 differential metabolites. 1: glycerophospholipid metabolism; 2: phenylalanine, tyrosine, and tryptophan biosynthesis; 3: phenylalanine metabolism; 4: linoleic acid metabolism; 5: *a*-linolenic acid metabolism; 6: arachidonic acid metabolism; 7: sphingolipid metabolism.

In this study, combining repeated compounds, a total of 138 differential metabolites were identified in seven groups. These identified differential metabolites belong to five families of compounds: lipids, amino acids, fatty acids, organic acids, and nucleosides. Lipids were detected in seven groups covering 20 lipid classes, namely, phosphatidylcholine (PC), phosphatidylethanolamine (PE), phosphatidylserine (PS), phosphatidylglycerol (PG), phosphatidylinositol (PI), phosphatidylglycerol phosphate (PGP), lysophosphatidylcholine (LysoPC), lysophosphatidylethanolamine (LysoPE), lysophosphatidylinositol (LysoPI), lysophosphatidic acid (LysoPA), sphingomyelin (SM), lysosphingomyelin (LysoSM), TG, monoacylglyceride (MG), diglyceride (DG), ceramide (Cer), lactosylceramide (LacCer), glucosylceramide (GlcCer), cholesteryl ester (CE), and sphinganine 1-phosphate (S1P). A total of 138 differential metabolites were imported into the MetPA website at one time for metabolic pathway analysis; the results are shown in Figure 11. Seven significant metabolic pathways of glycerophospholipid metabolism, sphingolipid metabolism, arachidonic acid metabolism, linoleic acid metabolism, phenylalanine metabolism, phenylalanine, tyrosine and tryptophan biosynthesis, and *a*-linolenic acid metabolism related to the 138 HUA differential metabolites were discovered.

The metabolites involved in glycerophospholipid metabolism and sphingolipid metabolism account for a large proportion of all metabolites, prompting attention to the correlation between abnormal lipid metabolism and HUA. In the previous study of HUA rat model by potassium oxonate or fructose in our laboratory [25, 40], glycerophospholipid metabolism was implicated. A large number of glycerophospholipids and their metabolites in serum samples of HUA patients were disturbed; phospholipid metabolism disorder occurred; PC, LysoPC, PE, LysoPE, PI, LysoPI, PS, PG, PGP, and LysoPA were mainly involved. Glycerophospholipid is a compound with the highest content in human body. It is an important component of biofilm and participates in cell membrane signal transduction and protein recognition. Among them, LysoPCs have a great influence on HUA and have been proved to increase the permeability of endothelial cells and affect the integrity of blood vessels under inflammatory conditions. For example, the downregulated levels of LysoPC(18 : 0), LysoPC(18 : 1(9Z)), and LysoPC(16 : 0) in HUA patients suggested a decrease in myocardial contractility and an increased probability of heart failure and myocardial infarction in HUA patients. LysoPCs are mainly metabolized in the liver and can significantly change in liver diseases and hepatotoxicity. The metabolic disorder of LysoPCs in serum is closely related to cirrhosis, fatty liver, viral hepatitis, and hepatitis B-associated liver cancer. SM, LysoSM, Cer, LacCer, GlcCer, and S1P were mainly involved in sphingolipid metabolism. These metabolites are involved in many important signal transduction processes, such as cell growth, differentiation, senescence, and death. Due to the disorders of glycerophospholipid metabolism and sphingolipid metabolism closely related to many diseases, such as metabolic syndrome, diabetes mellitus (DM), cardiovascular diseases (CVD), cerebrovascular diseases (CBVD), atherosclerosis, and cancer, indicating that with the increase of serum uric acid (SUA) levels, the risk of these diseases also increases. In addition, the transformation of metabolites involves the production of some inflammatory factors, which can cause inflammatory reactions in HUA patients.

Fatty acid metabolism is also involved in abnormal lipid metabolism; in this study, there are three pathways, namely, arachidonic acid metabolism, linoleic acid metabolism, and *a*-linolenic acid metabolism. In the previous study of the HUA rat model by fructose in our laboratory [40], arachidonic acid metabolism and linoleic acid metabolism were implicated. Linoleic acid can reduce lipids and cholesterol in the blood, soften blood vessels, regulate blood pressure, and accelerate blood circulation [41]. Linoleic acid is converted to *γ*-linolenic acid by 6-acyl-coenzyme A dehydrogenase in humans and mammals and further converted to arachidonic acid [42]. Arachidonic acid is closely related to lipid metabolism; its lipid-lowering effect is four times that of linoleic acid, which reduces the accumulation of fat in the body and lowers TG and LDL in blood lipids and raises HDL. Therefore, the reduction of arachidonic acid content will affect the regulation of lipid levels in the body. As shown in Table 1, compared with healthy people, the contents of TG and LDL in HUA patients were significantly increased, and the content of HDL was significantly decreased. Hypertriglyceridemia is the main cause of the onset of HUA. The ratio of serum LDL, TG, and TG to HDL was positively correlated with SUA levels, while HDL levels were inversely correlated with SUA levels. Arachidonic acid in cell membrane phospholipids can synthesize leukotrienes; it is associated with many inflammatory conditions, such as gout and arthritis. Α-Linolenic acid regulates fat storage, accelerates biological metabolism, and regulates the expression of inflammation-related genes. The fatty acids of palmitic acid and oleic acid are also involved in fatty acid metabolism, which have the effects of lowering blood glucose, regulating blood lipid levels, and reducing the risk of CVD [43, 44]. In a word, the disorder of multiple fatty acids suggests that HUA patients have a higher risk of CVD, CBVD, DM, skin diseases, and atherosclerosis. In the treatment of CVD and CBVD, DM, and hyperlipidemia, attention should be paid to the detection of SUA and prevention and treatment of HUA actively. Moreover, the metabolic disorder of unsaturated fatty acids in HUA patients is closely related to inflammation [45].

In this study, phenylalanine, tyrosine, and tryptophan were decreased in the serum of the HUA patient. Phenylalanine is an essential amino acid for the human body. Phenylalanine is converted into tyrosine by phenylalanine 4-hydroxylase, which together synthesize important neurotransmitters and hormones involved in the metabolism of glucose and fat [46, 47]. Phenylalanine is the upstream metabolic substance of tyrosine, and its content changes are closely related to tyrosine. The synthesis of dopamine and thyroid hormone, important neurotransmitters in the human body, requires the participation of tyrosine. Tyrosine can also be converted into fumaric acid and acetyl acetate to participate in metabolic activities, such as the TCA cycle, and provide energy for the body. Thyroid hormone is related to immunity and mainly plays a role in stabilizing metabolism in the body. Phenylalanine and tyrosine are also closely related to DM, hypertension, and other diseases [48, 49]. Tryptophan, under the action of indoleamine 2,3-dioxygenase, produces formylkynurenine, and then formylkynurenine rapidly converted into kynurenine by kynurenine formamidase, which is involved in inflammatory and immune responses.

## 5. Conclusions

In conclusion, in this work, the metabolomics approach based on UPLC-Q-TOF/MS was employed to investigate serum metabolic changes in the HUA patients; 138 potential differential metabolites related to HUA were identified, which provided associations of lipids, amino acids, fatty acids, organic acids, and nucleosides profiles with HUA individuals. Metabolic pathways involved in glycerophospholipid metabolism, sphingolipid metabolism, arachidonic acid metabolism, linoleic acid metabolism, phenylalanine metabolism, phenylalanine, tyrosine and tryptophan biosynthesis, and *a*-linolenic acid metabolism shed light on the understanding of the etiology and pathogenesis process of HUA.

## Figures and Tables

**Table 1:** Basic characteristics and biochemistry results of participants in the control group and HUA group.

Parameters	Control group (*n* = 20)	HUA group (*n* = 20)
Age (years)	41.1 ± 12.6	40.3 ± 11.6
Men (%)	55%	55%
FSG (mmol/L)	4.9 ± 0.4	5.8 ± 0.6^*∗*^
UA (*µ*mol/L)	320.4 ± 40.3	481.6 ± 51.0^*∗∗*^
TG (mmol/L)	1.52 ± 0.5	3.3 ± 1.2^*∗∗*^
ALT (U/L)	23.6 ± 10.3	40.7 ± 15.9^*∗∗*^
AST (U/L)	20.5 ± 5.7	27.7 ± 12.8^*∗*^
HDL (mmol/L)	1.5 ± 0.4	1.2 ± 0.2^*∗*^
LDL (mmol/L)	2.7 ± 0.6	3.6 ± 0.8^*∗∗*^
CR (*µ*mol/L)	79.5 ± 8.7	83.2 ± 14.7^*∗*^

*Note.* Continuous variables described as mean (standard deviation) and categorical variables as count (proportion). FSG: fasting serum glucose; UA: uric acid; TG: triglycerides; ALT: alanine aminotransferase; AST: aspartic aminotransferase; HDL: high-density lipoprotein cholesterol; LDL: low-density lipoprotein cholesterol; CR: creatinine. ^*∗*^ Significant difference from control at *p* < 0.05. ^*∗∗*^ Significant difference from control at *p* < 0.01.

**Table 2:** *R* ^2^ *Y* and *Q*^2^ value obtained for the seven groups of OPLS-DA score plots.

Parameters		A	B	C	D	E	F	G
ESI + mode	*R* ^*2*^ *Y*	0.846	0.716	0.870	0.843	0.969	0.909	0.918
	*Q* ^*2*^	0.728	0.593	0.793	0.755	0.817	0.840	0.862

ESI− mode	*R* ^*2*^ *Y*	0.895	0.990	0.985	0.989	0.951	0.898	0.990
	*Q* ^*2*^	0.650	0.766	0.698	0.790	0.726	0.761	0.724

## Data Availability

The data used to support the findings of this study are available from the corresponding author upon request.

## Supplementary Materials

Supplementary Materials Table S1: the differential metabolites identified of Group A. Table S2: the differential metabolites identified of Group B. Table S3: the differential metabolites identified of Group C. Table S4: the differential metabolites identified of Group D. Table S5: the differential metabolites identified of Group E. Table S6: the differential metabolites identified of Group F. Table S7: the differential metabolites identified of Group G.
