# Supplementary material for: High-Throughput Untargeted Serum Metabolomics Analysis of Hyperuricemia Patients by UPLC-Q-TOF/MS
Source: Evid Based Complement Alternat Med. 2021 Jun 12;2021:5524772. doi: 10.1155/2021/5524772 (PMC8216829; doi:10.1155/2021/5524772)
Supplement: Supplementary Materials — Table S1: the differential metabolites identified of Group A. Table S2: the differential metabolites identified of Group B. Table S3: the differential metabolites identified of Group C. Table S4: the differential metabolites identified of Group D. Table S5: the differential metabolites identified of Group E. Table S6: the differential metabolites identified of Group F. Table S7: the differential metabolites identified of Group G. [file 5524772.f1.docx]

**Supplementary File**

Nankun Qin,^1^ Yue Jiang,^1^ Wenjun Shi,^1^ Liting Wang, ^1^ Lingbo Kong, ^2^ Chengxiang Wang, ^1^ Yuying Guo, ^1^ Jiayu Zhang,^3^ and Qun Ma^1^

Table S1: The differential metabolites identified of the group A.

| NO. | Metabolites | Molecular Formula | VIP score | p-value | Fold change | Trend |
| --- | --- | --- | --- | --- | --- | --- |
| 1 | Tryptophan | C11H12N2O2 | 2.29833 | 5.73E-10 | −1.1324 | ↓ |
| 2 | Phenylalanine | C9H11NO2 | 2.51148 | 4.87E-10 | −1.1697 | ↓ |
| 3 | Valine | C5H11NO2 | 4.16052 | 2.19E-08 | −1.2122 | ↓ |
| 4 | Cer(d18:0/14:0) | C32H65NO3 | 10.2484 | 2.86E-08 | −2.9463 | ↓ |
| 5 | LysoPC(20:2(11Z,14Z)) | C28H54NO7P | 3.33295 | 1.32E-07 | −1.4099 | ↓ |
| 6 | Oleic acid | C18H34O2 | 3.21471 | 6.82E-07 | −1.4031 | ↓ |
| 7 | LysoPC(P-18:0/0:0) | C26H54NO6P | 2.40979 | 2.59E-06 | −1.3762 | ↓ |
| 8 | Arachidic acid | C20H40O2 | 16.5524 | 2.15E-06 | −1.0575 | ↓ |
| 9 | SM(d18:1/14:0) | C37H75N2O6P | 2.08734 | 0.000207 | −8.5503 | ↓ |
| 10 | SM(d18:0/22:0) | C45H93N2O6P | 5.30382 | 0.000226 | −9.2597 | ↓ |
| 11 | PG(18:0/18:1(9Z)) | C42H81O10P | 3.06194 | 0.000294 | −3.4134 | ↓ |
| 12 | PI(16:0/18:1(11Z)) | C43H81O13P | 2.23492 | 0.000346 | −9.8252 | ↓ |
| 13 | PS(16:0/18:0) | C40H78NO10P | 2.787 | 0.000214 | −27.42 | ↓ |
| 14 | PC(14:0/18:1(9Z)) | C40H78NO8P | 2.79734 | 0.000545 | −13.516 | ↓ |
| 15 | SM(d18:1/18:1(9Z)) | C41H81N2O6P | 2.69048 | 0.000688 | −15.576 | ↓ |
| 16 | LysoPC(18:1(9Z)) | C26H52NO7P | 1.97403 | 1.57E-09 | −1.2209 | ↓ |
| 17 | Cer(d18:0/26:0) | C44H89NO3 | 1.77216 | 4.01E-07 | −1.542 | ↓ |
| 18 | Arachidonic acid | C20H32O2 | 1.72948 | 7.33E-06 | −1.6144 | ↓ |
| 19 | PC(P-16:0/15:0) | C39H78NO7P | 1.5901 | 0.000163 | −27.489 | ↓ |
| 20 | Leucine | C6H13NO2 | 1.50181 | 4.87E-10 | −1.1697 | ↓ |
| 21 | LysoPC(16:0) | C24H50NO7P | 1.35352 | 8.16E-09 | −1.197 | ↓ |
| 22 | Tyrosine | C9H11NO3 | 1.48696 | 5.62E-08 | −1.0767 | ↓ |
| 23 | LysoPC(20:0/0:0) | C28H58NO7P | 1.18854 | 0.000199 | −14.306 | ↓ |
| 24 | LysoPC(18:0) | C26H54NO7P | 1.12942 | 4.28E-05 | −1.0864 | ↓ |
| 25 | PE(20:0/14:1(9Z)) | C39H76NO8P | 1.38705 | 0.000177 | −27.1 | ↓ |
| 26 | DG(20:0/24:0/0:0) | C47H92O5 | 1.36107 | 0.000192 | −7.4072 | ↓ |
| 27 | Palmitic acid | C16H32O2 | 1.04133 | 0.00062 | −1.1512 | ↓ |
| 28 | PE(18:0/18:1(9Z)) | C41H80NO8P | 1.25522 | 0.001038 | 1.2639 | ↑ |
| 29 | PGP(16:0/18:1(11Z)) | C40H78O13P2 | 1.49089 | 0.002677 | −8.0331 | ↓ |
| 30 | PS(18:0/18:1(9Z)) | C42H80NO10P | 1.1261 | 0.002826 | 1.5844 | ↑ |
| 31 | LysoPC(15:0) | C23H48NO7P | 3.90378 | 2E-05 | −2.5971 | ↓ |
| 32 | LysoPI(18:0/0:0) | C27H53O12P | 2.62849 | 0.000287 | 20.077 | ↑ |
| 33 | PE(14:0/14:0) | C33H66NO8P | 2.16284 | 0.003619 | 3.8544 | ↑ |
| 34 | LysoPC(14:1(9Z)) | C22H44NO7P | 2.85218 | 0.005703 | 2.9677 | ↑ |
| 35 | LysoPC(14:0) | C22H46NO7P | 2.27425 | 0.030451 | −2.0868 | ↓ |
| 36 | LysoPE(18:1(9Z)/0:0) | C23H46NO7P | 1.7213 | 0.005575 | 1.382 | ↑ |
| 37 | TG(12:0/12:0/12:0) | C39H74O6 | 1.06145 | 0.002044 | −1.0201 | ↓ |
| 38 | SM(d18:1/12:0) | C35H71N2O6P | 1.41545 | 0.01016 | 2.9978 | ↑ |

Note: ↑:Up-regulation, ↓: Down-regulation

Table S2: The differential metabolites identified of the group B.

| NO. | Metabolites | Molecular Formula | VIP score | p-value | Fold change | Trend |
| --- | --- | --- | --- | --- | --- | --- |
| 1 | Arachidic acid | C20H40O2 | 13.1858 | 1.59E-05 | −1.0112 | ↓ |
| 2 | DG(14:0/22:0/0:0) | C39H76O5 | 2.30626 | 5.56E-05 | 3.6364 | ↑ |
| 3 | SM(d18:1/22:1(13Z)) | C45H89N2O6P | 1.99915 | 8.44E-05 | 1.7155 | ↑ |
| 4 | DG(18:0e/2:0/0:0) | C23H46O4 | 1.54895 | 0.00095 | 1.6257 | ↑ |
| 5 | Cer(d18:0/22:0) | C40H81NO3 | 1.93603 | 0.001244 | 1.0997 | ↑ |
| 6 | LysoPC(18:2(9Z,12Z)) | C26H50NO7P | 1.84777 | 0.014978 | 1.1308 | ↑ |
| 7 | PG(18:0/18:0) | C42H83O10P | 2.57573 | 0.000729 | 1.1667 | ↑ |
| 8 | PS(16:0/18:0) | C40H78NO10P | 2.26725 | 2.22E-08 | −2.6948 | ↓ |
| 9 | Cer(d18:1/22:1(13Z)) | C40H77NO3 | 3.69478 | 0.000729 | 1.1667 | ↑ |
| 10 | SM(d18:0/22:0) | C45H93N2O6P | 5.21863 | 1.09E-06 | −1.842 | ↓ |
| 11 | PE(18:0/20:0) | C43H86NO8P | 1.6318 | 7.84E-09 | −1.8864 | ↓ |
| 12 | PC(15:0/22:1(13Z)) | C45H88NO8P | 1.30911 | 1.85E-08 | 3.0781 | ↑ |
| 13 | PGP(16:0/18:0) | C40H80O13P2 | 1.33153 | 7.77E-07 | −3.0917 | ↓ |
| 14 | PE(20:0/24:0) | C49H98NO8P | 1.31754 | 8.97E-07 | −3.5991 | ↓ |
| 15 | SM(d18:1/24:0) | C47H96N2O6P | 1.4756 | 0.000751 | 3.2784 | ↑ |
| 16 | LysoPE(0:0/24:1(15Z)) | C29H58NO7P | 5.69245 | 0.004997 | −1.5337 | ↓ |
| 17 | LysoPA(16:0/0:0) | C19H39O7P | 1.90087 | 0.00057 | −2.7212 | ↓ |
| 18 | PI(16:0/20:0) | C45H87O13P | 1.36938 | 0.000646 | −1.9946 | ↓ |
| 19 | PE(20:0/14:1(9Z)) | C39H76NO8P | 1.3655 | 0.0227 | −1.2793 | ↓ |
| 20 | SM(d18:1/12:0) | C35H71N2O6P | 1.02981 | 0.024132 | 2.1264 | ↑ |

Note: ↑:Up-regulation, ↓: Down-regulation

Table S3: The differential metabolites identified of the group C.

| NO. | Metabolites | Molecular Formula | VIP score | p-value | Fold change | Trend |
| --- | --- | --- | --- | --- | --- | --- |
| 1 | LysoPA(18:1(9Z)/0:0) | C21H41O7P | 4.04221 | 5.55E-14 | −1.3454 | ↓ |
| 2 | Phenylalanine | C9H11NO2 | 2.57038 | 1.64E-10 | −1.4347 | ↓ |
| 3 | Lactic acid | C3H6O3 | 8.18356 | 3.91E-12 | −1.3537 | ↓ |
| 4 | Valine | C5H11NO2 | 4.44122 | 4.51E-12 | −1.3295 | ↓ |
| 5 | LysoPC(16:0) | C24H50NO7P | 9.25691 | 1.34E-11 | −1.2346 | ↓ |
| 6 | LysoPC(18:2(9Z,12Z)) | C26H50NO7P | 9.56139 | 1.36E-11 | 1.0325 | ↑ |
| 7 | LysoPC(20:2(11Z,14Z)) | C28H54NO7P | 2.64082 | 7.09E-10 | −1.3366 | ↓ |
| 8 | Stearic acid | C18H36O2 | 3.36386 | 1.81E-09 | −1.4597 | ↓ |
| 9 | Arachidic acid | C20H40O2 | 18.5645 | 3.12E-09 | −1.9201 | ↓ |
| 10 | Oleic acid | C18H34O2 | 2.98013 | 2.28E-07 | −1.2907 | ↓ |
| 11 | Cer(d18:0/14:0) | C32H65NO3 | 7.97409 | 3.83E-06 | −1.3154 | ↓ |
| 12 | PE(14:0/18:2(9Z,12Z)) | C37H70NO8P | 2.41317 | 3.32E-05 | −2.5683 | ↓ |
| 13 | Tryptophan | C11H12N2O2 | 1.8515 | 1.64E-10 | −1.4347 | ↓ |
| 14 | Arachidonic acid | C20H32O2 | 1.51451 | 4.8E-06 | −1.0611 | ↓ |
| 15 | DG(18:0e/2:0/0:0) | C23H46O4 | 1.70778 | 2.7E-05 | 2.47 | ↑ |
| 16 | PC(16:0/15:0) | C39H78NO8P | 1.46086 | 2.21E-14 | −1.6171 | ↓ |
| 17 | LysoPC(18:1(9Z)) | C26H52NO7P | 1.67842 | 4.01E-13 | −1.4047 | ↓ |
| 18 | Leucine | C6H13NO2 | 1.2014 | 6.07E-10 | −1.1795 | ↓ |
| 19 | PE(15:0/P-16:0) | C36H72NO7P | 1.40464 | 1.17E-07 | −1.5054 | ↓ |
| 20 | LysoPE(0:0/14:1(9Z)) | C19H38NO7P | 1.411 | 0.0017 | −1.8461 | ↓ |
| 21 | DG(24:0/15:0/0:0) | C42H82O5 | 1.37035 | 2.77E-06 | −2.1626 | ↓ |
| 22 | Linoleic acid | C18H32O2 | 1.24667 | 4.41E-06 | −1.4039 | ↓ |
| 23 | MG(0:0/16:0/0:0) | C19H38O4 | 1.18588 | 6.96E-06 | −2.3827 | ↓ |
| 24 | Palmitic acid | C16H32O2 | 1.39006 | 0.000311 | −1.021 | ↓ |
| 25 | PE(14:0/P-16:0) | C35H70NO7P | 1.27233 | 0.000133 | −1.1313 | ↓ |
| 26 | PI(16:0/16:0) | C41H79O13P | 3.57578 | 0.001129 | −4.0175 | ↓ |

Note: ↑:Up-regulation, ↓: Down-regulation

Table S4: The differential metabolites identified of the group D.

| NO. | Metabolites | Molecular Formula | VIP score | p-value | Fold change | Trend |
| --- | --- | --- | --- | --- | --- | --- |
| 1 | PC(16:0/P-18:0) | C42H84NO7P | 2.30427 | 4E-14 | −1.281 | ↓ |
| 2 | CE(24:1(15Z)) | C51H90O2 | 3.32591 | 3.56E-11 | −1.0265 | ↓ |
| 3 | Cer(d18:0/26:0) | C44H89NO3 | 5.4595 | 3.59E-11 | −1.3792 | ↓ |
| 4 | LysoPA(18:0e/0:0) | C21H45O6P | 7.11979 | 7.49E-09 | 2.0244 | ↑ |
| 5 | LysoPC(P-18:0) | C26H54NO6P | 8.26893 | 0.026222 | −1.3199 | ↓ |
| 6 | LysoPC(O-18:0) | C26H56NO6P | 8.41569 | 1.91E-06 | −1.15 | ↓ |
| 7 | MG(22:0/0:0/0:0) | C25H50O4 | 2.50632 | 5.35E-05 | −1.6221 | ↓ |
| 8 | PG(16:0/16:0) | C38H75O10P | 3.19231 | 0.005629 | −2.2367 | ↓ |
| 9 | PE(P-16:0e/0:0) | C21H44NO6P | 2.15552 | 1.55E-12 | −1.558 | ↓ |
| 10 | S1P | C18H40NO5P | 1.99605 | 4.08E-07 | −1.3909 | ↓ |
| 11 | LysoPC(16:0) | C24H50NO7P | 1.70187 | 1.15E-05 | −1.0979 | ↓ |
| 12 | TG(16:0/16:0/16:0) | C51H98O6 | 1.70205 | 0.000121 | −1.8337 | ↓ |
| 13 | Linoleic acid | C18H32O2 | 1.99754 | 0.001136 | −1.1819 | ↓ |
| 14 | MG(0:0/24:1(15Z)/0:0) | C27H52O4 | 1.836 | 3.65E-12 | −2.1532 | ↓ |
| 15 | LysoPC(22:2(13Z,16Z)) | C30H58NO7P | 1.34129 | 0.036059 | 1.2449 | ↑ |
| 16 | alpha-Linolenic acid | C18H30O2 | 1.73626 | 0.010332 | −2.5784 | ↓ |
| 17 | LysoSM(d18:1) | C23H50N2O5P | 3.45626 | 4.1E-06 | 2.833 | ↑ |
| 18 | LysoPA(16:0/0:0) | C19H39O7P | 2.19456 | 0.000243 | −1.5515 | ↓ |
| 19 | LysoPI(18:0/0:0) | C27H53O12P | 6.66209 | 0.000641 | 2.1058 | ↑ |
| 20 | SM(d17:1/24:0) | C46H94N2O6P | 2.00136 | 0.000701 | 1.183 | ↑ |
| 21 | PS(18:0/18:1(9Z)) | C42H80NO10P | 2.4499 | 0.002963 | 1.1793 | ↑ |
| 22 | LysoPC(15:0) | C23H48NO7P | 3.16553 | 0.00937 | −2.2922 | ↓ |
| 23 | LysoPC(14:0) | C22H46NO7P | 5.65043 | 0.014341 | −2.38 | ↓ |
| 24 | TG(8:0/8:0/8:0) | C27H50O6 | 2.5085 | 1.75E-05 | −1.0078 | ↓ |
| 25 | PC(14:0/18:1(11Z)) | C40H78NO8P | 1.72554 | 0.007079 | 1.3538 | ↑ |
| 26 | LysoPA(0:0/16:0) | C19H39O7P | 2.86574 | 7.65E-05 | −5.83 | ↓ |
| 27 | PE(24:0/14:1(9Z)) | C43H84NO8P | 1.44619 | 0.006602 | 1.3227 | ↑ |
| 28 | LysoPC(P-18:1(9Z)) | C26H52NO6P | 1.95253 | 0.008547 | −2.4881 | ↓ |

Note: ↑:Up-regulation, ↓: Down-regulation

Table S5: The differential metabolites identified of the group E.

| NO. | Metabolites | Molecular Formula | VIP score | p-value | Fold change | Trend |
| --- | --- | --- | --- | --- | --- | --- |
| 1 | TG(16:0/16:0/16:0) | C51H98O6 | 4.15272 | 1.59E-11 | −1.2519 | ↓ |
| 2 | Linoleic acid | C18H32O2 | 2.67108 | 9.37E-10 | −1.2206 | ↓ |
| 3 | LysoPC(15:0) | C23H48NO7P | 3.12773 | 4.46E-08 | −1.2967 | ↓ |
| 4 | PE(14:0/15:0) | C34H68NO8P | 3.92749 | 2.74E-07 | −2.0564 | ↓ |
| 5 | LysoPC(16:0) | C24H50NO7P | 13.5382 | 1.32E-06 | −1.1048 | ↓ |
| 6 | LysoPC(22:1(13Z)) | C30H60NO7P | 2.92693 | 1.62E-05 | −1.1778 | ↓ |
| 7 | LysoPC(6:0) | C14H30NO7P | 2.42804 | 5.95E-05 | −1.2116 | ↓ |
| 8 | Palmitic acid | C16H32O2 | 2.46823 | 0.00035 | −1.0316 | ↓ |
| 9 | Arachidonic acid | C20H32O2 | 1.80457 | 7.42E-10 | −1.2266 | ↓ |
| 10 | S1P | C18H40NO5P | 1.84535 | 2.32E-07 | −1.2605 | ↓ |
| 11 | SM(d18:0/23:0) | C46H95N2O6P | 1.76589 | 7.35E-07 | −1.2945 | ↓ |
| 12 | LysoPC(22:2(13Z,16Z)) | C30H58NO7P | 1.40343 | 0.00124 | 1.377 | ↑ |
| 13 | LysoPC(P-18:0) | C26H54NO6P | 1.40807 | 0.002162 | −1.9677 | ↓ |
| 14 | LysoPC(16:1(9Z)) | C24H48NO7P | 1.36267 | 0.007407 | 1.5738 | ↑ |
| 15 | LysoPA(16:0/0:0) | C19H39O7P | 3.19623 | 0.000548 | −2.526 | ↓ |
| 16 | PE(18:0/18:1(9Z)) | C41H80NO8P | 4.24484 | 0.003299 | 1.4867 | ↑ |
| 17 | PE(18:0/18:0) | C41H82NO8P | 3.02021 | 0.005212 | 1.6242 | ↑ |
| 18 | PC(14:0/24:0) | C46H92NO8P | 2.20336 | 0.005325 | 1.1155 | ↑ |
| 19 | PE(14:0/24:1(15Z)) | C43H84NO8P | 3.3054 | 0.005372 | 1.3967 | ↑ |
| 20 | PC(15:0/18:1(11Z)) | C41H80NO8P | 5.50287 | 0.00608 | 1.958 | ↑ |
| 21 | PC(14:0/16:1(9Z)) | C38H74NO8P | 8.33003 | 0.006732 | 1.3822 | ↑ |
| 22 | PE(14:0/20:1(11Z)) | C39H76NO8P | 8.1868 | 0.006984 | 1.9952 | ↑ |
| 23 | PI(18:0/18:0) | C45H87O13P | 5.77171 | 0.015212 | 2.0773 | ↑ |
| 24 | PC(14:0/16:0) | C38H76NO8P | 3.05708 | 0.015775 | 1.0697 | ↑ |
| 25 | PE(15:0/20:1(11Z)) | C40H78NO8P | 4.00545 | 0.018161 | 1.9512 | ↑ |
| 26 | PC(18:1(9Z)/18:0) | C44H86NO8P | 2.93252 | 0.034855 | 1.1377 | ↑ |
| 27 | LysoPA(18:1(9Z)/0:0) | C21H41O7P | 2.66533 | 0.000162 | −2.1606 | ↓ |
| 28 | PS(18:0/18:1(9Z)) | C42H80NO10P | 2.24927 | 0.003458 | 1.9159 | ↑ |
| 29 | PC(18:0/20:1(11Z)) | C46H90NO8P | 3.52507 | 0.00025 | 1.093 | ↑ |
| 30 | LysoPI(18:0/0:0) | C27H53O12P | 2.61005 | 4.61E-05 | 2.4963 | ↑ |
| 31 | CE(16:1(9Z)) | C43H74O2 | 1.86882 | 0.000508 | 1.0151 | ↑ |
| 32 | LysoPC(14:0) | C22H46NO7P | 1.66247 | 0.003341 | −1.0018 | ↓ |
| 33 | PC(15:0/16:1(9Z)) | C39H76NO8P | 2.12733 | 0.011914 | 2.1335 | ↑ |

Note: ↑:Up-regulation, ↓: Down-regulation

Table S6: The differential metabolites identified of the group F.

| NO. | Metabolites | Molecular Formula | VIP score | p-value | Fold change | Trend |
| --- | --- | --- | --- | --- | --- | --- |
| 1 | LysoPA(18:1(9Z)/0:0) | C21H41O7P | 6.08938 | 1.01E-05 | −1.3187 | ↓ |
| 2 | Phenylalanine | C9H11NO2 | 2.78795 | 8.82E-05 | −2.7355 | ↓ |
| 3 | LysoPC(18:2(9Z,12Z)) | C26H50NO7P | 14.0139 | 1.09E-11 | 1.2681 | ↑ |
| 4 | Lactic acid | C3H6O3 | 3.6712 | 2.11E-11 | −1.0631 | ↓ |
| 5 | Valine | C5H11NO2 | 5.7341 | 3.68E-11 | −1.299 | ↓ |
| 6 | Linoleic acid | C18H32O2 | 2.55507 | 5.19E-10 | −1.5545 | ↓ |
| 7 | Oleic acid | C18H34O2 | 3.98314 | 6.4E-07 | −1.0735 | ↓ |
| 8 | Palmitic acid | C16H32O2 | 2.98999 | 8.71E-07 | −1.2523 | ↓ |
| 9 | LysoPC(6:0) | C14H30NO7P | 2.16634 | 0.000104 | −1.2575 | ↓ |
| 10 | SM(d18:1/24:1(15Z)) | C47H93N2O6P | 2.0887 | 0.004819 | 1.1119 | ↑ |
| 11 | Arachidic acid | C20H40O2 | 4.25671 | 0.007564 | −1.3053 | ↓ |
| 12 | Stearic acid | C18H36O2 | 3.13147 | 0.012549 | −1.6309 | ↓ |
| 13 | LysoPE(0:0/14:1(9Z)) | C19H38NO7P | 2.24154 | 4.38E-13 | −1.0534 | ↓ |
| 14 | PGP(18:0/18:1(9Z)) | C42H82O13P2 | 1.97061 | 4.91E-13 | −1.0213 | ↓ |
| 15 | TG(16:0/16:0/16:0) | C51H98O6 | 1.48619 | 4.57E-12 | −1.2997 | ↓ |
| 16 | PC(24:0/15:0) | C48H96NO8P | 1.74056 | 9.48E-12 | −1.1423 | ↓ |
| 17 | LysoPC(18:0) | C26H54NO7P | 1.88144 | 1.14E-11 | −1.1071 | ↓ |
| 18 | LysoPC(18:1(9Z)) | C26H52NO7P | 1.51112 | 5.87E-11 | −1.1542 | ↓ |
| 19 | Tyrosine | C9H11NO3 | 1.58783 | 1.46E-10 | −1.021 | ↓ |
| 20 | Arachidonic acid | C20H32O2 | 1.58306 | 1.02E-09 | −1.4292 | ↓ |
| 21 | LysoPC(16:0) | C24H50NO7P | 1.62512 | 6.18E-08 | −1.192 | ↓ |
| 22 | LysoPC(0:0/16:0) | C24H50NO7P | 2.01797 | 7.64E-08 | −1.019 | ↓ |
| 23 | SM(d18:0/23:0) | C46H95N2O6P | 1.46801 | 4.98E-07 | −1.3635 | ↓ |
| 24 | S1P | C18H40NO5P | 1.5971 | 1.7E-06 | −1.1455 | ↓ |
| 25 | DG(20:0/24:0/0:0) | C47H92O5 | 1.49902 | 1.98E-06 | −1.3318 | ↓ |
| 26 | SM(d17:1/24:1(15Z)) | C46H92N2O6P | 2.16403 | 1.91E-05 | 1.9118 | ↑ |
| 27 | SM(d18:1/24:0) | C47H96N2O6P | 1.9569 | 0.000173 | 2.5204 | ↑ |
| 28 | DG(15:0/24:0/0:0) | C42H82O5 | 1.44697 | 9.75E-12 | −1.1409 | ↓ |
| 29 | LysoPC(15:0) | C23H48NO7P | 1.29575 | 0.000187 | −1.7005 | ↓ |
| 30 | Cer(d18:1/24:1(15Z)) | C42H81NO3 | 1.34863 | 0.000824 | 1.3369 | ↑ |
| 31 | PC(14:0/24:1(15Z)) | C46H90NO8P | 1.94963 | 0.003138 | 1.5763 | ↑ |
| 32 | Leukotriene A4 | C20H30O3 | 1.33102 | 0.003631 | −1.7816 | ↓ |
| 33 | MG(0:0/15:0/0:0) | C18H36O4 | 2.06305 | 0.003746 | −3.0977 | ↓ |
| 34 | GlcCer(d18:1/12:0) | C36H69NO8 | 1.41797 | 0.013124 | 2.8237 | ↑ |
| 35 | Cer(d18:1/18:0) | C36H71NO3 | 2.16102 | 0.001693 | −2.1523 | ↓ |
| 36 | PE(14:0/20:1(11Z)) | C39H76NO8P | 6.709 | 0.006357 | 1.3623 | ↑ |
| 37 | PC(14:0/16:1(9Z)) | C38H74NO8P | 6.14134 | 0.008144 | 1.0341 | ↑ |
| 38 | PE(18:0/18:1(9Z)) | C41H80NO8P | 4.38119 | 0.009214 | 1.3239 | ↑ |
| 39 | LysoPA(18:0e/0:0) | C21H45O6P | 2.05829 | 0.020908 | 1.1425 | ↑ |
| 40 | LysoPI(18:0/0:0) | C27H53O12P | 2.04075 | 0.041788 | 1.0048 | ↑ |
| 41 | SM(d18:1/20:0) | C43H87N2O6P | 2.25154 | 0.003392 | 1.1331 | ↑ |
| 42 | LysoPC(22:1(13Z)/0:0) | C30H60NO7P | 1.08008 | 0.000974 | 2.9992 | ↑ |
| 43 | PC(P-18:1(9Z)/14:0) | C40H78NO7P | 1.38696 | 0.001079 | 2.0902 | ↑ |
| 44 | LysoPC(0:0/18:0) | C26H54NO7P | 1.14061 | 0.046167 | −1.1783 | ↓ |
| 45 | SM(d17:1/24:0) | C46H94N2O6P | 1.13664 | 0.001268 | 3.371 | ↑ |
| 46 | SM(d18:1/18:0) | C41H84N2O6P | 1.68419 | 0.001283 | 1.8463 | ↑ |
| 47 | SM(d18:0/16:0) | C40H74NO10P | 1.38543 | 0.001597 | 1.3575 | ↑ |
| 48 | SM(d18:1/22:0) | C45H91N2O6P | 1.06529 | 0.002505 | 3.3661 | ↑ |
| 49 | PE(20:0/14:1(9Z)) | C39H76NO8P | 1.43738 | 0.004792 | −4.1959 | ↓ |
| 50 | PC(15:0/16:1(9Z)) | C39H76NO8P | 1.30086 | 0.013716 | 1.6536 | ↑ |

Note: ↑:Up-regulation, ↓: Down-regulation

Table S7: The differential metabolites identified of the group G.

| NO. | Metabolites | Molecular Formula | VIP score | p-value | Fold change | Trend |
| --- | --- | --- | --- | --- | --- | --- |
| 1 | Phenylalanine | C9H11NO2 | 2.75047 | 3.14E-15 | −1.1956 | ↓ |
| 2 | DG(18:0/24:0/0:0) | C45H88O5 | 5.09791 | 1.89E-14 | −2.46 | ↓ |
| 3 | LysoPA(18:1(9Z)/0:0) | C21H41O7P | 4.86745 | 1.93E-13 | −1.2778 | ↓ |
| 4 | PC(15:0/20:0) | C43H86NO8P | 4.68529 | 5.06E-13 | −1.6664 | ↓ |
| 5 | Lactic acid | C3H6O3 | 2.73743 | 5.82E-12 | −1.3149 | ↓ |
| 6 | PS(18:0/20:0) | C44H86NO10P | 3.57358 | 8.32E-12 | −2.4771 | ↓ |
| 7 | LysoPC(16:0) | C24H50NO7P | 11.6888 | 5.7E-11 | −1.0402 | ↓ |
| 8 | LysoPA(16:0/0:0) | C19H39O7P | 2.61545 | 1.57E-10 | −1.2608 | ↓ |
| 9 | LysoPC(28:1(5Z)) | C36H72NO7P | 2.69873 | 1.73E-09 | −1.0117 | ↓ |
| 10 | Oleic acid | C18H34O2 | 3.63473 | 4.4E-09 | −1.062 | ↓ |
| 11 | PE(24:0/20:0) | C49H98NO8P | 2.69801 | 1.15E-08 | −2.521 | ↓ |
| 12 | Palmitic acid | C16H32O2 | 2.63392 | 1.15E-07 | −1.0686 | ↓ |
| 13 | SM(d18:1/24:0) | C47H96N2O6P | 3.18409 | 2.5E-07 | 13.508 | ↑ |
| 14 | PC(14:0/24:1(15Z)) | C46H90NO8P | 2.86389 | 1.15E-06 | 1.1479 | ↑ |
| 15 | SM(d17:1/24:1(15Z)) | C46H92N2O6P | 4.24345 | 2.59E-06 | 5.9274 | ↑ |
| 16 | MG(18:0/0:0/0:0) | C21H42O4 | 17.5169 | 4.13E-06 | −1.5062 | ↓ |
| 17 | SM(d18:0/22:0) | C45H93N2O6P | 6.23278 | 5.48E-06 | −2.295 | ↓ |
| 18 | SM(d18:1/24:1(15Z)) | C47H93N2O6P | 7.21914 | 2.93E-05 | 1.9845 | ↑ |
| 19 | PC(15:0/22:1(13Z)) | C45H88NO8P | 2.47157 | 3.81E-05 | 2.5073 | ↑ |
| 20 | Stearic acid | C18H36O2 | 5.58158 | 0.002077 | −2.1858 | ↓ |
| 21 | PE(20:0/20:0) | C45H90NO8P | 2.0087 | 0.023325 | −1.6108 | ↓ |
| 22 | PE(18:0/24:0) | C47H94NO8P | 2.09363 | 3.64E-11 | −2.5746 | ↓ |
| 23 | LacCer(d18:1/12:0) | C42H79NO13 | 1.98319 | 2.17E-06 | 4.5552 | ↑ |
| 24 | LysoPC(0:0/16:0) | C24H50NO7P | 1.91805 | 1.14E-15 | −1.2353 | ↓ |
| 25 | LysoPC(P-18:1(9Z)) | C26H52NO6P | 1.57656 | 3.08E-13 | −1.3309 | ↓ |
| 26 | DG(20:0/24:0/0:0) | C47H92O5 | 1.59736 | 7.78E-12 | −3.0046 | ↓ |
| 27 | Coenzyme A | C21H36N7O16P3S | 1.75984 | 5.04E-07 | 9.2921 | ↑ |
| 28 | Linoleic acid | C18H32O2 | 1.7744 | 6.45E-07 | −1.3253 | ↓ |
| 29 | LysoPC(O-18:0) | C26H56NO6P | 2.36665 | 1.03E-06 | −1.5427 | ↓ |
| 30 | LysoPC(16:1(9Z)) | C24H48NO7P | 2.01994 | 1.41E-06 | 1.0087 | ↑ |
| 31 | PC(15:0/24:0) | C47H94NO8P | 1.67792 | 3.53E-05 | 5.5277 | ↑ |
| 32 | DG(14:0/20:0/0:0) | C37H72O5 | 1.54946 | 0.0014 | −1.2232 | ↓ |
| 33 | Cer(d18:1/16:0) | C34H67NO3 | 4.83964 | 0.000122 | 1.0974 | ↑ |
| 34 | PC(14:0/16:1(9Z)) | C38H74NO8P | 4.48785 | 0.003194 | 1.0791 | ↑ |
| 35 | LysoPC(15:0) | C23H48NO7P | 4.24159 | 0.005221 | −1.6895 | ↓ |
| 36 | SM(d18:0/18:1(11Z)) | C41H83N2O6P | 1.65605 | 0.001904 | 1.1624 | ↑ |
| 37 | PE(P-18:0/15:0) | C38H76NO7P | 1.45602 | 0.000304 | 2.2219 | ↑ |
| 38 | SM(d18:1/20:0) | C43H87N2O6P | 1.33891 | 0.000735 | 1.2493 | ↑ |
| 39 | SM(d18:0/22:1(13Z)) | C45H91N2O6P | 1.15371 | 0.010933 | 1.1002 | ↑ |
| 40 | LysoPA(18:0e/0:0) | C21H45O6P | 1.14273 | 0.042723 | 1.2259 | ↑ |

Note: ↑:Up-regulation, ↓: Down-regulation
